# NeuroMeasure: A Software Package for Quantification of Cortical Motor Maps Using Frameless Stereotaxic Transcranial Magnetic Stimulation

**DOI:** 10.3389/fninf.2019.00023

**Published:** 2019-04-16

**Authors:** Michael B. Gerber, Alasdair C. McLean, Samuel J. Stephen, Alex G. Chalco, Usman M. Arshad, Gary W. Thickbroom, Josh Silverstein, K. Zoe Tsagaris, Amy Kuceyeski, Kathleen Friel, Taiza E. G. Santos, Dylan J. Edwards

**Affiliations:** ^1^Biomedical Engineering Department, The City College of New York, New York, NY, United States; ^2^Burke Neurological Institute, White Plains, NY, United States; ^3^Department of Radiology, Brain and Mind Research Institute, Weill Cornell Medicine, New York, NY, United States; ^4^Brain Mind Research Institute, Weill Cornell Medicine, New York, NY, United States; ^5^Blythedale Children's Hospital, Valhalla, NY, United States; ^6^Department of Neurosciences and Behavioral Sciences, Ribeirao Preto Medical School, University of São Paulo, Ribeirao Preto, São Paulo, Brazil; ^7^Moss Rehabilitation Research Institute, Elkins Park, PA, United States; ^8^Edith Cowan University, Joondalup, WA, Australia

**Keywords:** transcranial magnetic stimulation, brain mapping, software package, neuroimaging, motor cortex

## Abstract

The recent enhanced sophistication of non-invasive mapping of the human motor cortex using MRI-guided Transcranial Magnetic Stimulation (TMS) techniques, has not been matched by refinement of methods for generating maps from motor evoked potential (MEP) data, or in quantifying map features. This is despite continued interest in understanding cortical reorganization for natural adaptive processes such as skill learning, or in the case of motor recovery, such as after lesion affecting the corticospinal system. With the observation that TMS-MEP map calculation and quantification methods vary, and that no readily available commercial or free software exists, we sought to establish and make freely available a comprehensive software package that advances existing methods, and could be helpful to scientists and clinician-researchers. Therefore, we developed *NeuroMeasure*, an open source interactive software application for the analysis of TMS motor cortex mapping data collected from Nexstim® and BrainSight®, two commonly used neuronavigation platforms. NeuroMeasure features four key innovations designed to improve motor mapping analysis: de-dimensionalization of the mapping data, fitting a predictive model, reporting measurements to characterize the motor map, and comparing those measurements between datasets. This software provides a powerful and easy to use workflow for characterizing and comparing motor maps generated with neuronavigated TMS. The software can be downloaded on our github page: https://github.com/EdwardsLabNeuroSci/NeuroMeasure

**Aim:**

This paper aims to describe a software platform for quantifying and comparing maps of the human primary motor cortex, using neuronavigated transcranial magnetic stimulation, for the purpose of studying brain plasticity in health and disease.

## Introduction

There is ongoing interest in characterizing the cortical representation of limb muscles in humans from the TMS-motor evoked potential. Motor map reorganization is considered to occur in relation to motor learning from practice, and with recovery from brain lesion. These phenomena are supported by increasing number of studies in animal models such as the rodent, and non-human primates. Yet, animal models typically have a stimulation specificity advantage, such as with optogenetics in the rodent (Lim et al., 2013), or intracortical microstimulation in the monkey (Nudo and Milliken, 1996). Human invasive (electrical) stimulation is also possible, however transcranial magnetic stimulation (TMS) has practical (non-invasive) and physiological (no anesthesia required) advantages. What remains contentious with TMS however, is that the spatial specificity of the motor maps is ambiguous, and thus interpretation of the data is limited. For example, a crude but reasonable method to establish map area historically, is to sum the scalp sites (typically 1 cm spacing) from where a response following TMS was observed (Wassermann et al., 1992; Melgari et al., 2008). Responsive sites are flanked by areas where TMS was delivered, but a response was not observed. Note that this is “scalp-representation,” not “cortical representation,” since the stimulation sites were on the scalp. Others have interpolated the mean MEP amplitude data such that smooth continuous contours representing the spatial representation of MEP amplitude change across sites (Borghetti et al., 2008), then defined non-zero map edges. A limitation of this method is that the validity of the map fitting has not been sufficiently interrogated experimentally, and the map is confined to the scalp rather than the cortex. TMS using neuronavigation has allowed the electrical field to be projected onto the cortical anatomy below the stimulation site, with greater consistency and precision for coil position (and orientation, hence e-field trajectory). Two commonly used and commercially available systems for TMS neuronavigation are Brainsight™ and Nexstim™. Both have been used for TMS motor mapping. In the case of Nexstim™, the United States Food and Drug Administration (FDA) has approved the device for motor mapping to aid with neurosurgical planning (FDA Regulation Number: 21 CFR 882.5805). In each of these systems, the cortical target corresponding to the center of the projected electric field is assigned the motor evoked potential amplitude recorded using limb muscle surface electromyography (sEMG). The potential spatial error is in the order of millimeters (Wassermann et al., 1992; Kleim et al., 2007). This method can establish the approximate boundary of muscle representations of the cortex, and has useful application to examine the relationship to cortical topography and structures/foci of clinical relevance.

For assessing map change with time, the neuroscience field has developed the practice of sampling the motor cortex and representing motor evoked potentials over a grid space, where the TMS pulses are applied (Kleim et al., 2007; Littmann et al., 2014). Comparisons are then made between maps by directly comparing the values of likewise points on the grid. While effective, the standardization of the grid space makes data comparison between maps that were not collected under those standards impossible. Few attempts have been made in the field to employ mathematical model fitting to estimate values between the collected data points and generate a function that can be sampled anywhere for a predicted value (Wilson et al., 1993; Arya et al., 2010).

We developed a workflow based on model fitting for motor map quantification, and comparison, and have made this software available to facilitate research into its applicability for investigational and clinical use. The models lend themselves to computations of surface area and volume integral. Furthermore, the particular model fitting algorithms included in this package are non-parametric algorithms developed for creating smooth interpolations with high goodness-of-fit. This is convenient for motor mapping data, as the underlying distribution of TMS-evoked responses from the motor cortex are not fully understood, so a non-parametric model without such assumptions is ideal. The advantage of this approach is that comparisons between maps is simpler, as the motor map function can be sampled at any location and compared with its likewise point from another motor map.

While there are many ways for a motor map to be constructed, the most common methodology involves the stimulation of sites on the motor cortex in a grid via TMS (Wassermann et al., 1992; Kleim et al., 2007; Jonker et al., 2018). Electrodes placed on the skin over a target muscle record MEPs during the stimulation procedure and every position on the sampling grid can then be assigned a value based on the excitability of the corticospinal pathway to the target muscle. This value is typically reported as the peak-to-peak amplitude of the EMG response (measured in microvolts or millivolts), but can also be latency between time of stimulation and time of response (measured in milliseconds). In the present manuscript, we only consider the peak-to-peak magnitude, which is the most common mapping parameter.

**Figure 1:**
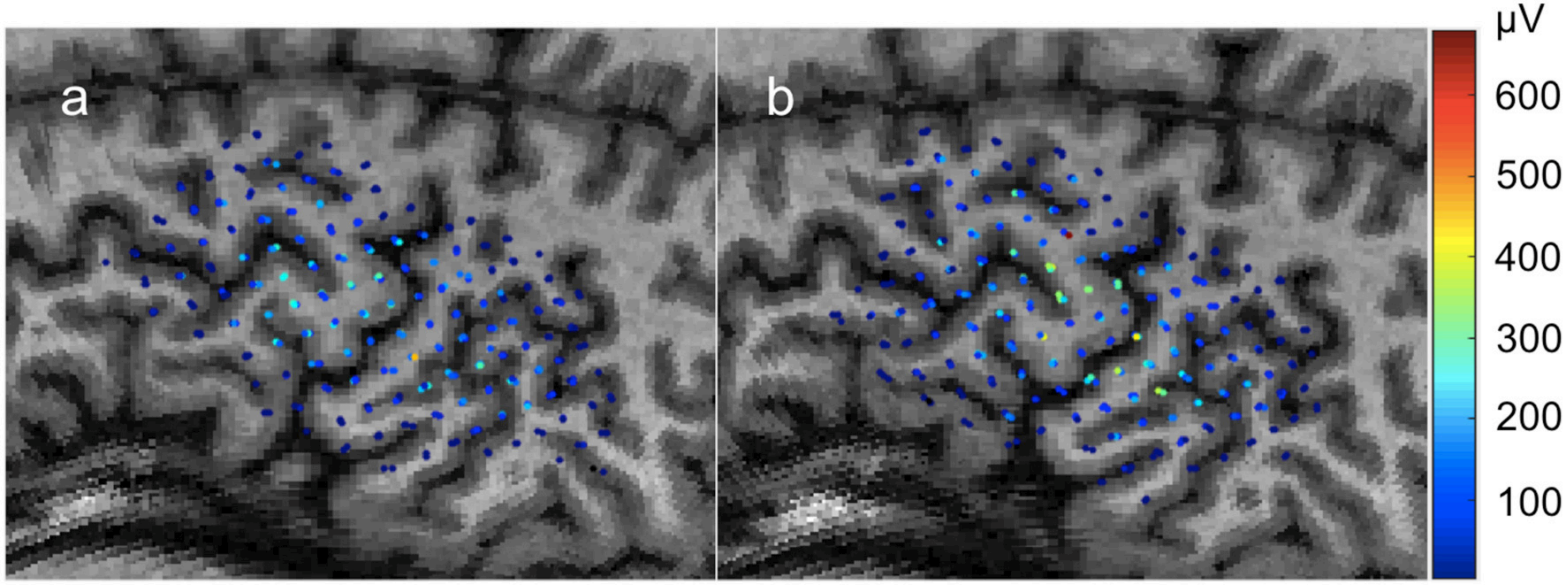
Individual motor evoked potential amplitudes sampled three times per location in a 0.5 × 0.5cm grid. Color represents peak-to-peak MEP amplitude in μV. Recorded from the FDI muscle with TMS. **(a)** First recording. **(b)** Second recording begun immediately after the conclusion of the first with the same parameters.

The sampling grid can be thought of as 2D coordinate system conformed over the curvature of the scalp (Kohl et al., 2006). This grid space is registered to a patient's MRI and is often exported from integrated motor mapping system as 3D coordinates with respect to an anatomical landmark, or to the origin of the MRI's 3D image array. In summary, every position in the grid space has an X (left to right), Y (posterior to anterior), and Z (inferior to superior) associated with a value that is the peak-to-peak magnitude of a MEP elicited when stimulating that location. An example of a typical motor map is illustrated in Figure 1, and some examples of a motor map's features, are listed briefly in Table 1. The center of gravity (COG) in particular is commonly used to make comparisons as it describes both shifts in position and amplitude of the MEP field (Guerra et al., 2015). However, motor evoked potential (MEP) amplitude at a given stimulation site and intensity, is variable across stimuli (Thickbroom et al., 1998), in part due to random, spontaneous fluctuations in corticospinal and segmental motoneuron excitability levels (Kiers et al., 1993), and state-dependent fluctuations (Abbruzzese et al., 1996) in corticospinal excitability, while the contribution from unstable coil position and trajectory are controversial (Jung et al., 2010). Various methods have been reported to reduce MEP variance, yet variability in health and disease remains an important consideration (Schambra et al., 2015). Using custom mapping software, Thickbroom and colleagues accounted for MEP variance and showed acceptable reproducibility in TMS-generated MEP motor maps, with a 1–2 mm variation in center of gravity over 30 repetitions in a sample size of 5 healthy subjects (Thickbroom et al., 1998). Contemporary methods of data fitting for generating maps should suitably account for MEP variability.

**Table 1:** Summary of map features calculated by NeuroMeasure.

**Measurement**	**Summary**	**Units**
Center of Gravity (COG)	Weighted average of measurement positions where the weights are the values of the measurements.	Distance (mm)
Peak Amplitude	Position of the absolute maximum measurement within the mapping field.	Distance (mm)
Surface Area (SA)	The summation of areal patches along the map whose MEP value are above a set threshold.	Distance^2^ (mm^2^)
Volume Integral (VI)	The summation of volumetric patches. A volumetric patch is the product between an areal patch and its corresponding MEP value.	Intensity^*^distance^2^ (μv^*^mm^2^)
Root Mean Square Error (RMSE)	The average difference between all sets of likewise values between two motor maps.	Intensity (μv)
Area Under the Curve (AUC)	The performance of a predictive model fitted onto dataset 1 in predicting dataset 2.	Unitless

While TMS hardware has evolved significantly since its debut to make it the versatile and robust stimulation method it is today, with notable advancements in coil type, field strength, spatial resolution and navigation, and stimulation technique (e.g., repetitive TMS and paired-pulse TMS) (Rossini et al., 2015), a platform for performing standardized and easily accessible analysis of this data is still not available. Such a platform would need to report map features and provide a convenient workflow for comparing these map features between datasets collected from the same patient. Thus, the purpose of this design project was to create such a software platform, offering researchers and clinicians an easy to use workflow for quantifying and comparing motor maps generated with neuronavigated TMS.

In the present paper, we present the *NeuroMeasure* software platform for quantifying and comparing TMS-generated maps of motor cortex that may be used in the study of motor plasticity, such as in response to biological recovery or therapeutic treatments after damage affecting the human corticospinal system.

## Software Design

The software is open source and can be downloaded on our github page: https://github.com/EdwardsLabNeuroSci/NeuroMeasure

The NeuroMeasure software comes with detailed installation instructions and a user's guide highlighting the operation of the app is also downloadable on the github linked above.

## Overview

The need addressed by this software package is quantification and temporal comparison of motor maps generated by the Nexstim and BrainSight integrated motor mapping systems. The process for achieving this involves three steps. First we convert the 3D Cartesian coordinates of the exported motor map to 2D angular coordinates in order to reduce the number of independent variables while losing minimal positional information. Then, we fit a non-parametric 2D surface function to the 2D angular motor map. Finally, we compute the center of gravity and maximum value from the raw input data, and compute surface area and volume integral of the fitted surface function. These metrics can be compared between motor maps generated from the same patient, along with a visual overlay of the value differences at likewise positions in angular space, as long as the two datasets were generated with respect to the same reference point (or were aligned).

## Workflow

**Figure 2:**
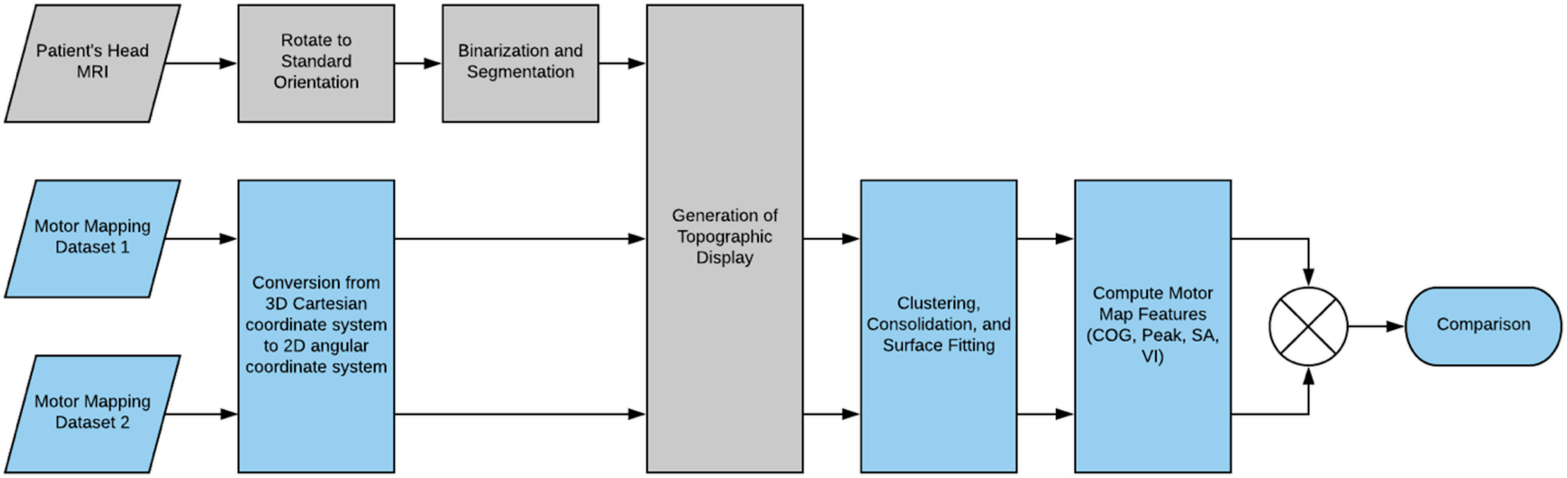
Flowchart of the NeuroMeasure workflow.

Here, we present a basic outline of the application's workflow, summarized in Figure 2. First, the MRI scan of the patient's head that has been registered to the TMS recording session is uploaded to the app. The scan is reoriented to a standard orientation used by Nexstim and BrainSight, and segmented to generate a binary mask separating the head from the background. The binary mask is used to slice the 3D image to generate a globe-like topographic projection of the head and its anatomical features. Now, the motor mapping data is uploaded to the application and positional data is converted from 3D Cartesian coordinates to 2D angular coordinates which are readily mapped to the topographic display. A reference point is also uploaded together with the dataset as a means of standardizing coordinate systems between datasets. The data points can be clustered to group repeated-measurements if necessary, and they can be fitted to a 2D surface function from one of several surface fitting algorithms. Computation of the center of gravity and maximum value were possible prior to surface fitting, but now surface area and volume integral of the surface function can also be computed to further characterize the motor map. Furthermore, the fitted surface function facilitates the comparison of likewise points between datasets as any coordinate on the 2D angular plane can be sampled for a predicted MEP value that can be compared to its likewise position on another dataset.

## Key Innovations

### Data Dimensionality and Topographic Display

The first major consideration of the software's design was about the dimensionality of motor mapping data. A TMS coil applied to the brain produces a magnetic field with a distribution that can be approximated with simulation. This resultant distribution is paired with a close approximation of the location and direction of the stimulus using a process called “neuronavigation,” which is automatically performed in both the BrainSight and Nexstim systems when registering MEP's to the 3D coordinates of a patient's MRI scan (Lüdemann-Podubecká and Nowak, 2016). Typically a figure eight coil is used because the resultant electric field generated using a coil of this geometry is much more focused directly underneath the center of the coil as opposed to the wide dispersal pattern seen in single ring coils. From the center of the figure eight coil, field strength tapers off as distance from the center of stimulation increases. The MEP value is then assigned to the position of the peak magnetic field strength, but it is important to understand that the region of the brain under influence of the magnetic field is not a just that point, but includes the region around that point. It would be errant to conclude that the resultant MEP location is the location of a particular neuronal strand responsible for innervation; rather, we assume in the design of the software that the combination of induced neurons surrounding the MEP location are responsible ultimately for signaling muscle contraction.

Despite the values being mapped to 3D coordinates, for our purposes of fitting a predictive model, the grid is effectively 2D. The coil is always applied to the surface of the scalp and the depth of the effective stimulus of the magnetic field is fixed to 1–4 cm so the resulting data points take the shape of a 2D sheet that conforms to the scalp. Since data points don't vary in depth, there is no need to interpolate the values of MEP's in 3D and we can simplify the predictive modeling problem to 2D, which saves considerable computational resources.

$$\begin{array}{l} x_{o}=\frac{\overset{N}{\underset{i=1}{\sum }}x_{i}}{N}y_{o}=\frac{\overset{N}{\underset{i=1}{\sum }}y_{i}}{N}z_{o}=\frac{\overset{N}{\underset{i=1}{\sum }}z_{i}}{N} \end{array}$$

Equation 1: x_o_, y_o_, z_o_ are the coordinates of the centroid. x_i_, y_i_, z_i_ are the indexed coordinates of the points in the point cloud. N is the total number of points in the point cloud.

**Figure 3:**
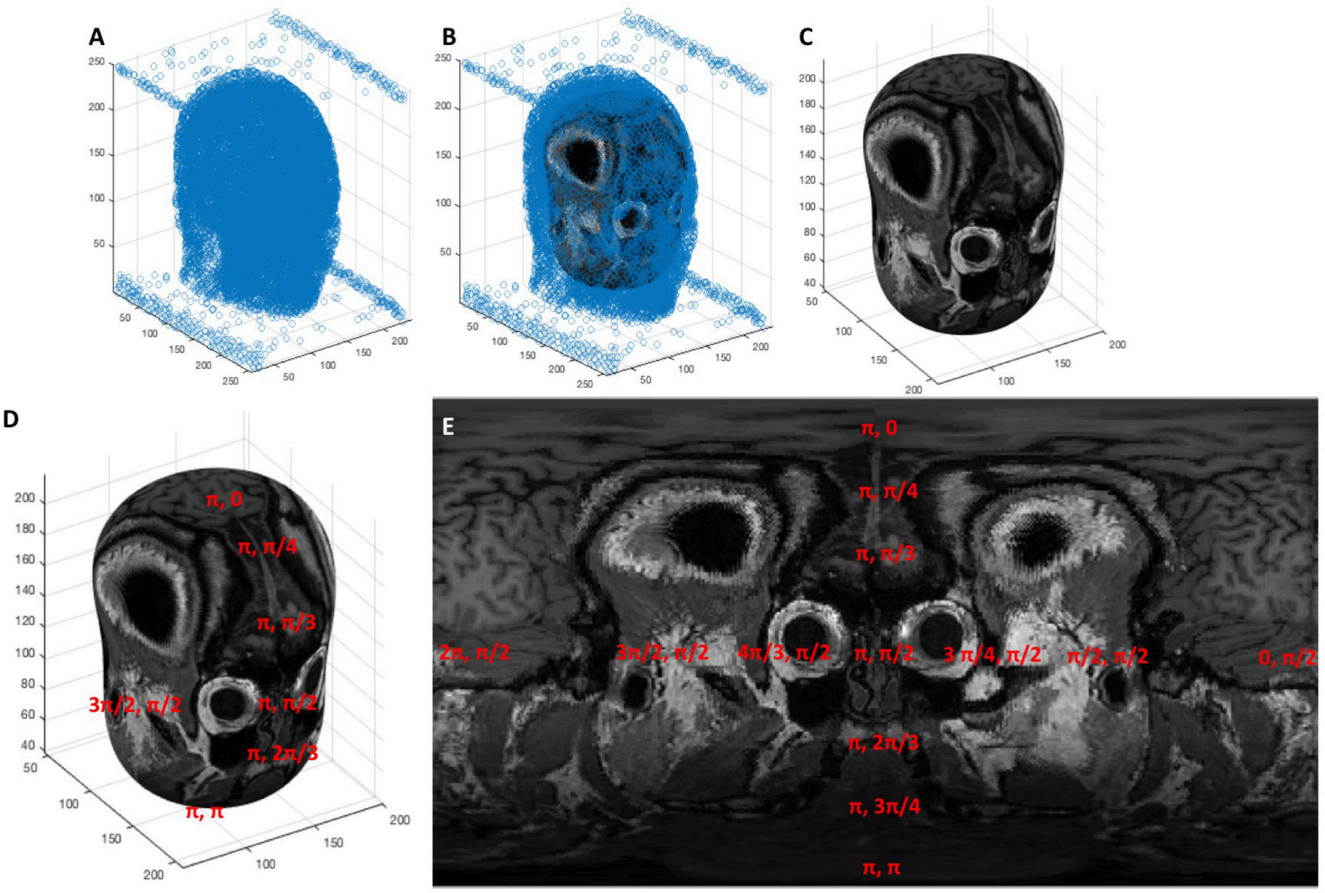
**(A)** A point cloud generated from sampling the segmentation of the head every 25th voxel, **(B)** an ellipsoid fit to the point cloud via least squares regression, **(C)** the ellipsoid shown without the point cloud surrounding it, **(D,E)** the elliptic surface position is described by an angular coordinate system that, when projected onto an image, creates the topographic display.

The most desirable strategy for de-dimensionalizing the data would be such that the Euclidean distance between any two points in 3D Cartesian space and in the new 2D space would be proportional. Loss of this proportionality leads to data distortion, causing data at the extremes to appear shrunken compared to their true distances and sizes. Since the data fits the scalp which approximates the shape of a sphere we found that conversion to spherical coordinates without an “r” dimension, such that all 3D points are fit onto a spherical shell, produces less distortion than z-squashing where the z dimension is effectively ignored in the 3D Cartesian system. We refer to this spherical coordinate system without an “r” dimension as angular coordinates. An important consideration for the conversion between angular and Cartesian coordinates is the origin, which must be at the center of curvature of the data in order to minimize distortion. We approximate the center of curvature of the data points to be the geometric centroid of the head, which we compute using a segmented binary mask of the head generated from the patient's imported scan. We convert this mask into a point cloud by sampling every 25th “white” voxel in the binarized image volume shown in Figure 3. The centroid of this point cloud is than computed via Equation 1. The data points are then converted from 3D Cartesian coordinates into angular coordinates from the centroid using Equation 2.

$$\begin{array}{l} \theta_{i}=\text{co}{\text{s}}^{-1}\left(\frac{\left(z_{i}-z_{o}\right)}{\sqrt{{\left(x_{i}-x_{o}\right)}^{2}+{\left(y_{i}-y_{o}\right)}^{2}+{\left(z_{i}-z_{o}\right)}^{2}}}\right)\\ \phi_{i}=\text{ta}{\text{n}}^{-1}\left(\frac{\left(y_{i}-y_{o}\right)}{\left(x_{i}-x_{o}\right)}\right) \end{array}$$

Equation 2: θ and ϕ are the azimuth and inclination angle, respectively. x_i_,y_i_,z_i_ are the coordinates of the point cloud and x_o_, y_o_, z_o_ are the coordinates of the centroid.

Now that our data points are converted to angular coordinates, we would also like to map them to the anatomical features obtained from the MRI scan and display the two concurrently. To represent a 3D image volume in an angular coordinate space, we must generate spherical image slices. We found that the best topographic displays of the cortex were generated from ellipsoid shapes fitted to approximate the shape of the scalp instead of pure spheres. Thus, we fit an ellipsoid to the head segmentation discussed previously and use that geometry to section our 3D image volume and generate image slices that display the motor cortex in a “globe-like” projection.

First, we manipulate the point cloud that we use for our centroid calculation in the same way as our data and convert it to angular coordinates via Equation 2. We now use a least squares regression to fit a function that relates angular coordinates, expressed as (θ,ϕ), to 3D Cartesian coordinates, expressed as (x,y,z). The function was chosen to be that of an ellipsoid with a radial term represented by a fourth order polynomial, shown in Equation 3, converted into matrix format in Equation 4, and the weights solved for in Equation 5. We found experimentally that 10 parameters was the cutoff range where further parameters no longer benefitted the qualitative shape of the ellipsoid to approximate the shape of the scalp.

$$\begin{array}{l} x-x_{o}=w_{1}+(w_{2}+w_{3}\theta +w_{4}\phi +w_{5}\theta^{2}+w_{6}\phi^{2}+w_{7}\theta^{3}\\ \;+w_{8}\phi^{3}+w_{9}\theta^{4}+w_{10}\phi^{4})\text{ cos}\left(\theta \right)\text{ sin}\left(\phi \right)\\ y-y_{o}=w_{1}+(w_{2}+w_{3}\theta +w_{4}\phi +w_{5}\theta^{2}+w_{6}\phi^{2}+w_{7}\theta^{3}\\ \;+w_{8}\phi^{3}+w_{9}\theta^{4}+w_{10}\phi^{4})\text{ sin}\left(\theta \right)\text{ sin}\left(\phi \right)\\ z-z_{o}=w_{1}+(w_{2}+w_{3}\theta +w_{4}\phi +w_{5}\theta^{2}+w_{6}\phi^{2}+w_{7}\theta^{3}\\ \;+w_{8}\phi^{3}+w_{9}\theta^{4}+w_{10}\phi^{4})\text{ cos}\left(\phi \right) \end{array}$$

Equation 3: θ and ϕ are the azimuth and inclination angle, respectively. x_i_,y_i_,z_i_ are the coordinates of the point cloud and x_o_, y_o_, z_o_ are the coordinates of the centroid.

The solved weights from Equation 5 can then be substituted back into Equation 3 to compute the equivalent 3D Cartesian coordinates from any set of angular coordinates. This establishes a means by which 3D spatial position can be registered onto a 2D gridspace and projected into an image. This process is shown visually in Figure 3, where a point cloud (Figure 3A) representing the segmentation of a head is fitted to an ellipsoid (Figures 3B,C) whose surface is characterized by an angular coordinate system (Figure 3D). The MRI scan can then be sampled by reading the grayscale voxel position at every angular position on the ellipsoid's surface, which can then be represented as a 2D topographic map shown in Figure 3E.

a
  $$\begin{array}{l} a\left[\begin{array}{c} x_{i}-x_{o}\\ y_{i}-y_{o}\\ z_{i}-z_{o} \end{array}\right]=\left[\begin{array}{ccc} w_{x1} & w_{y1} & w_{z1}\\ w_{x2} & w_{y2} & w_{z2}\\ w_{x3} & w_{y3} & w_{z3}\\ w_{x4} & w_{y4} & w_{z4}\\ w_{x5} & w_{y5} & w_{z5}\\ w_{x6} & w_{y6} & w_{z6}\\ w_{x7} & w_{y7} & w_{z7}\\ w_{x8} & w_{y8} & w_{z8}\\ w_{x9} & w_{y9} & w_{z9}\\ w_{x10} & w_{y10} & w_{z10} \end{array}\right]\\ \;\left[\begin{array}{c} 1\\ 1\\ 1 \end{array}\left(\begin{array}{ccccccccc} 1 & \theta_{i} & \phi_{i} & {\theta_{i}}^{2} & {\phi_{i}}^{2} & {\theta_{i}}^{3} & {\phi_{i}}^{3} & {\theta_{i}}^{4} & {\phi_{i}}^{4}\\ 1 & \theta_{i} & \phi_{i} & {\theta_{i}}^{2} & {\phi_{i}}^{2} & {\theta_{i}}^{3} & {\phi_{i}}^{3} & {\theta_{i}}^{4} & {\phi_{i}}^{4}\\ 1 & \theta_{i} & \phi_{i} & {\theta_{i}}^{2} & {\phi_{i}}^{2} & {\theta_{i}}^{3} & {\phi_{i}}^{3} & {\theta_{i}}^{4} & {\phi_{i}}^{4} \end{array}\right)\begin{array}{c} \text{ cos}\left(\theta_{i}\right)\text{ sin}\left(\phi_{i}\right)\\ \text{ sin}\left(\theta_{i}\right)\text{ sin}\left(\phi_{i}\right)\\ \text{ cos}\left(\phi_{i}\right) \end{array}\right] \end{array}$$
b
  $$\begin{array}{l} \left[\begin{array}{l} x_{i}-x_{o}\\ y_{i}-y_{o}\\ z_{i}-z_{o} \end{array}\right]=\left[\begin{array}{lll} w_{x} & w_{y} & w_{z} \end{array}\right]\left[\begin{array}{l} I_{x}\\ I_{y}\\ I_{z} \end{array}\right] \end{array}$$

Equation 4: (a) The same equation from equation 3 substituted for (x_i_,y_i_,z_i_) the 3D Cartesian coordinates of the point cloud and their angular equivalents (θ_i_,ϕ_i_) and converted into matrix format, (b) the same equation from (a) abbreviated with substitutions.

**Figure 4:**
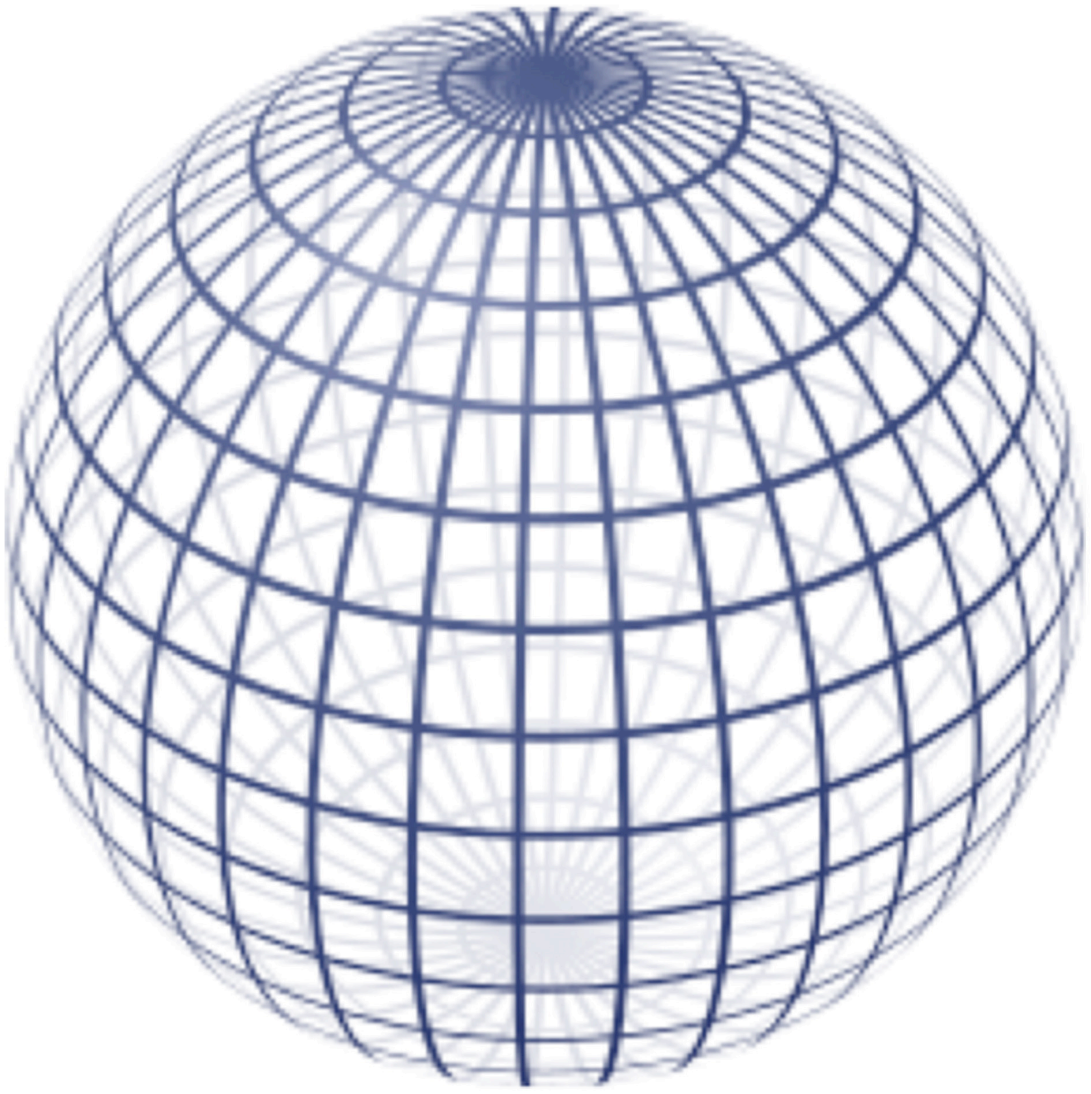
A representation of how the angular coordinate system is mapped onto the surface of a sphere. © 2009 Geek3 / GNU-FDL, commons.wikimedia.org/wiki/File:Sphere_wireframe_10deg_10r.svg.

The loss of proportionality between 3D Cartesian distance and angular distance, or distortion, mentioned previously is further evident in the topographic map at the poles where the features appear “stretched.” To better understand how our de-dimensionalization strategy leads to distortion, consider the more simplified geometry of a sphere shown in Figure 4. The gridlines show how angular coordinates describe every position on the sphere's surface. The longitude and latitude lines represent increments of θ and ϕ, respectively. Note how as the latitude increases toward the pole, the arc length between two longitude lines gets smaller until they eventually converge onto a singularity at the pole. At this singularity, the coordinates (π,0), (2π/3,0), (π/4,0) all represent different positions on the 2D topographic map, but the same 3D point on the ellipsoid's surface. In fact, this is true everywhere on the map to some extent; the proportionality between angular dimensions and 3D arc length is not conserved throughout the map and thus there will always be some misrepresentation of distance depending on what proportion is used as the reference (in our case, the equator). This is the reason why the topographic map appears “distorted” toward its extremes. Because surface fitting also relies on this conversion, it has implications regarding the interpolated motor map and measurements that rely on it like surface area and volume integral. We correct for this distortion to the best of our ability by snapping the center of gravity of the data points to the “equator” of the topographic map to minimize the distortion at the poles as much as possible, however better solutions to this problem are a focus of future development.

$$\begin{array}{l} \left[\begin{array}{c} w_{x}\\ w_{y}\\ w_{z} \end{array}\right]=\left[\begin{array}{c} \left({{I^{\prime }}_{x}}^{*}I_{x}\right)\backslash \left({{I^{\prime }}_{x}}^{*}\left(x_{i}-x_{o}\right)\right)\\ \left({{I^{\prime }}_{y}}^{*}I_{y}\right)\backslash \left({{I^{\prime }}_{y}}^{*}\left(y_{i}-y_{o}\right)\right)\\ \left({{I^{\prime }}_{z}}^{*}I_{z}\right)\backslash \left({{I^{\prime }}_{z}}^{*}\left(z_{i}-z_{o}\right)\right) \end{array}\right] \end{array}$$

Equation 5: Solving for the parametric weights using the standard form of the least squares regression formula. Note the slash notation (\) represents a left to right matrix division.

### Clustering and Surface Fitting

Since our software is intended to be a research tool for studying neuroplasticity, an important consideration was its capability to support a number of different experimental formats. Particularly, we found that repeated measures experiments were prominent in the literature (Freundlieb et al., 2015; Lüdemann-Podubecká and Nowak, 2016; Potter-Baker et al., 2016) and added features to cluster repeated measures into groups, consolidate the clusters into single-point representations, and finally fit the data representation onto a predictive model in the form of a surface function. The clustering itself is done with a quality threshold clustering algorithm that uses normalized distance between points as a means of grouping (Danalis et al., 2012). The user selects a threshold in the range of 0–1 where 0 clusters no points and thus each value is considered a separate measurement event, and 1 clusters all the points into one group. The user is expected to manually choose a threshold that groups the points in a desired manner such that points that are intended to be repeated measures at the same position are grouped, while separate measurement events remain separate.

#### Cluster Consolidation

Now that repeated measurements have been clustered, the subset can be consolidated to a singular, representative value.

There are five methods for cluster consolidation that can be performed on each node to prepare the dataset for model fitting. The current operations are *averaging, maximum, minimum, variance*, and *probability*. By selecting the *averaging* operation, for example, the cluster is consolidated to a single point whose value is represented by the mean of the values in the cluster. The *maximum* and *minimum* operations will similarly set the value of the single point representative to the maximum or the minimum of the grouped values, respectively. Notably, the units of the data points generated from *average, maximum*, and *minimum* clustering consolidation remains as the unit used in the imported data (if imported values are in units of μV, the resulting units are in μV). Selecting the *variance* operation will use the variance formula shown in equation 6 to compute the variance of the grouped points. The units of variance are the units of the imported values squared (if imported values are in units of μV, the resulting units are in μV^2^).

$$\begin{array}{l} s=\frac{\overset{N}{\underset{i=1}{\sum }}{\left(v_{i}-\mu \right)}^{2}}{N} \end{array}$$

Equation 6: The variance formula, where s is the variance, N is the total number of points in the cluster, v_i_ is the i^th^ value of the points in the cluster and μ is the mean of the cluster. Variance is the average of squared differences between each individual value of the grouped points and the overall mean.

Finally, the *probability* operation is a special case that is different from the four other cluster consolidation methods. Unlike the last four methods, which kept the data in its raw and continuous numerical format, the probability operation will binarize the values so that they are categorical. When selecting *probability*, the software will request the input of a binarization threshold, which is set to 40 μV by default. The raw dataset is binarized with this threshold so that all values above it become represented as 1, and all values below it become represented as 0. Then, a “probability score” is computed for each cluster depending on how many values within the group are above or below the threshold. For example, if a cluster contains four values, and two of those values are above the threshold (and therefore calculated as 1), and two values are below the threshold (and therefore calculated as 0), the overall probability value assigned to the cluster is 50%, because two of the values have exceeded the specified threshold. Further, if there are three values in a cluster and two are 1's and one is a 0, the overall probability is 66.67%. In single measurement experiments where there is only one value per cluster, the data points will always be either 0 or 100% depending on whether they were above or below the set threshold. The units of the resulting consolidated data, when generated from the probability operation, are in unitless percentage. The value of using the probability option is in understanding general trends (as percentage indicates the likelihood that a stimulus will successfully elicit a sufficient response) or in comparison with other motor maps. The chosen threshold will greatly affect the results of the model fitting. Currently, there are no recommendations for setting the binarization threshold, as the inclusion of this feature into NeuroMeasure is experimental and is intended largely for research rather than an absolute deterministic answer. Table 2 summarizes the different cluster consolidation methods and their resulting units.

**Table 2:** Summary of all available cluster consolidation methods and their resulting units.

**Operation**	**Summary**	**Output data type**
Average	The representative value of the cluster is equal to the mean of the clustered measurements.	μV
Maximum	The representative value of the cluster is equal to the absolute maximum of the clustered measurements.	μV
Minimum	The representative value of the cluster is equal to the absolute minimum of the clustered measurements.	μV
Variance	The representative value of the cluster is equal to the variance of the clustered measurements, as computed via equation 6.	μV^2^
Probability	The representative value of the cluster is equal to the probability of evoking an action potential higher than a pre-set binarization threshold. This is computed by applying the binarization threshold to the subset of clustered. measurements and averaging the resulting binary values. Example: If there are three values in a cluster, three 1's averages to 1 (100%), two 1's and one 0 averages to 0.66 (66%), and one 1 and two 0's averages to 0.33 (33%).	Unitless (%)

#### Surface Fitting Algorithms

Surface fitting is the process of fitting a continuous function of the form z = S(x,y), to a set of known data points (x,y,z), that uses two independent variables x and y to predict the dependant variable z. In our case, x and y are the two angular coordinates θ and ϕ, and z is the value assigned to the clusters of a repeated measures experiment (average MEP, minimum MEP, maximum MEP, variance of MEPs, or probability of response) or a flat MEP value in the case of single-measurement experiments.

The application currently supports four diverse non-parametric surface fitting methods to yield a fitted surface function from the post-clustered data points. The algorithms, being non-parametric, mean they do not assume that the underlying data is sampled from a normal distribution and they only attempt to provide a high goodness-of-fit regardless of the map's “shape”; this was deemed ideal for motor mapping data where the expected distribution is not well understood. Piecewise Cubic uses a bi-cubic function as the basis of interpolation. The cubic algorithm fits a different function between every three data points and connects all the patches to produce a single curve/surface. Piecewise Linear utilizes a similar algorithm but uses a bi-linear function as opposed to bi-cubic. Biharmonic (v4) is a fourth-order partial differential equation using the bilaplacian (biharmonic) operator, which is the square of the Laplacian operator. Unlike the piecewise cubic and piecewise linear algorithms that can produce either curves or surfaces depending on the data, biharmonic is only designed to yield a surface. Locally weighted scatterplot smoothing (lowess) method can smooth data through locally weighted linear regression. The piecewise cubic algorithm is recommended for most cases as it generates the best goodness of fit, however it is recommended to see the operation manual on our Github for a delineation of the pros and cons between different fit algorithms based on experimentation. The surface fitting algorithms are summarized in Table 3. Also, see the Mathworks website's curve fitting section for a detailed description of the different surface fitting algorithms available in NeuroMeasure: https://www.mathworks.com/help/curvefit/interpolation-methods.html#bsz6baz

**Table 3:** Summary of surface fitting algorithms available in NeuroMeasure.

**Algorithm**	**Summary**
Piecewise cubic spline	3rd order 2D polynomial fit to dataset in patches of 3 data points each, fit in a piecewise fashion directly without least squares. Edges between patches are smoothed by splines.
Local linear Weighted scatter Smoothing; a.k.a Lowess	1st order 2D polynomial fit to dataset via least squares regression combined with a ratio that splits the dataset into local parts and the polynomial is fit in a piecewise fashion. Ratio controls the degree of smoothing. Here, the ratio is fixed to 0.25 (0 = least smooth, 1 = most smooth)
Biharmonic (v4)	The same algorithm used in MATLAB's griddata function. See the comprehensive explanation on the Mathworks website griddata documentation: https://www.mathworks.com/help/matlab/ref/griddata

### Measurements

Measurements of the motor map features are the most important part of the application and one of the end goals that our workflow is designed to accomplish. Fundamentally, the system produces four measurements from the dataset: the center of gravity (COG), the peak value, the map's surface area, and its volume integral. COG is a classic means of representing motor mapping data, typically used to compare motor maps before and after a stimulus meant to initiate neuroplasticity (Wilson et al., 1993; Thickbroom et al., 1998; Guerra et al., 2015; van de Ruit and Grey, 2017). The peak value of the map is also relatively simple and similar to COG, although it is not used as extensively. Neither peak nor COG require the generation of a fitted surface function to generate. The fitted surface function can, however, add to the repertoire of available tools by supplying a surface area over which the motor map elicits above a certain MEP value threshold, or a volume integral computed similarly to surface area but with the addition of the z-dimension. All are discussed in further detail below.

Notably, COG and peak position are reported in x,y,z dimensions of the original 3D Cartesian coordinate system in which the data points were uploaded, as well as Euclidean distance with respect to a reference point to facilitate comparison with other motor maps.

#### Reference Point

**Figure 5:**
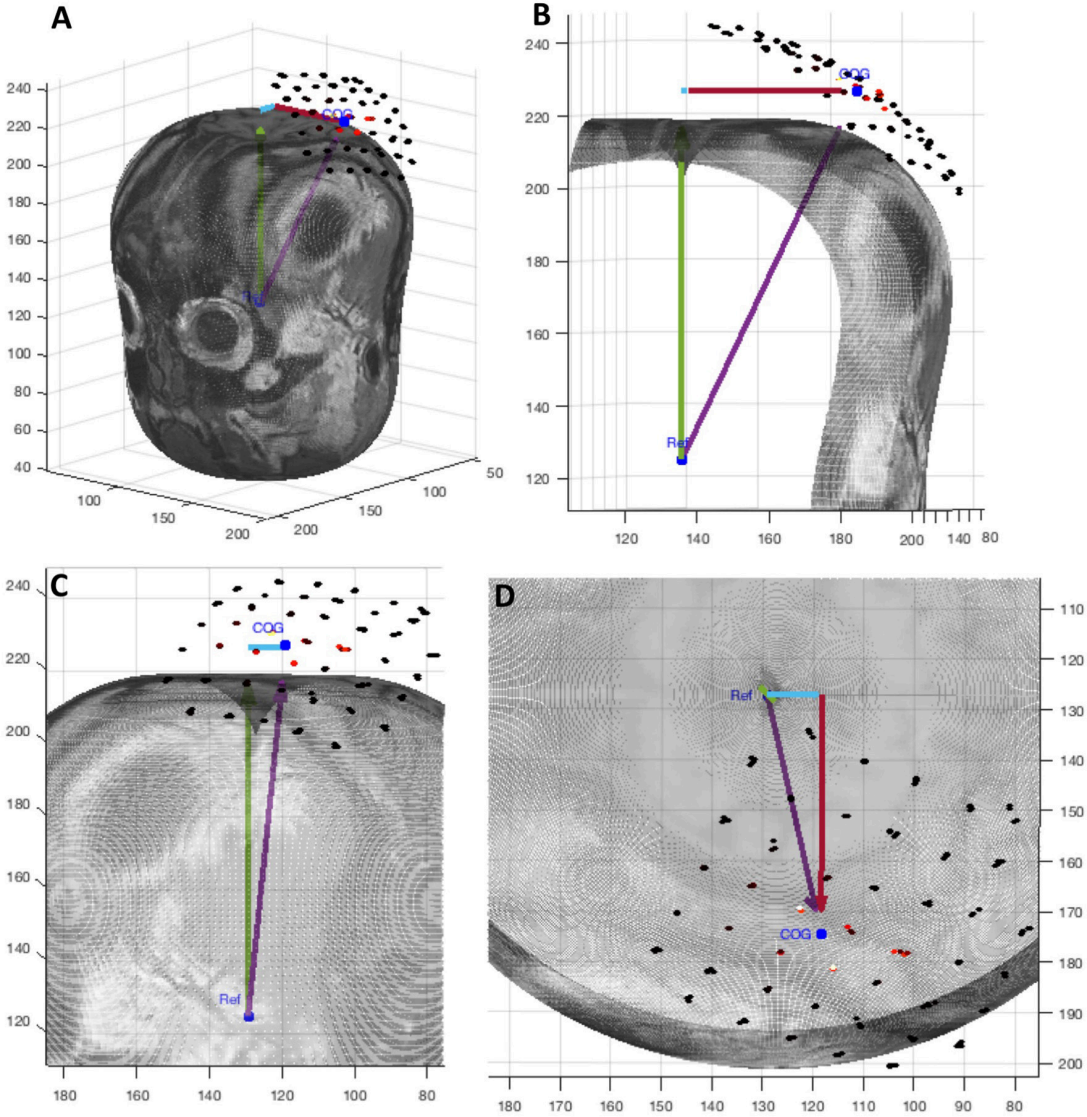
A graphic display of the four position measurements reported in NeuroMeasure's position table. The cyan arrow represents posterior->anterior distance, red arrow represents right->left distance, green arrow represents inferior->superior distance and the purple arrow represents Euclidean distance. The 3D slice is shown from different view angles: **(A)** isometric, **(B)** coronal, **(C)** sagittal, **(D)** transverse.

All measurements of position in NeuroMeasure are reported with respect to a reference point chosen by the user. This allows for direct measurement comparisons from a standardized anatomical reference point between maps generated at different time points. The reference point can be imported along with the dataset from Nexstim or BrainSight neuronavigation systems. If the user does not enter a reference point, it is set to the centroid of the head segmentation by default (Figure 5). The reference point can also be changed interactively within the app.

#### Center of Gravity

The center of gravity is a well-established point of interest (Thickbroom et al., 1998) for characterizing a motor map because of its convenient condensation of the values and positions of many data points into one. Although referred to as center of gravity in the neuroscience literature (Thickbroom et al., 1998), it should be noted that the engineering and mathematics literature refers to this measurement as center of mass due to it being a feature of a scalar field as opposed to a vector field. Here, we will continue to call it center of gravity in accordance with neuroscience convention.

$$\begin{array}{l} X_{COG}=\frac{\overset{N}{\underset{i=1}{\sum }}\left(x_{i}•v_{i}\right)}{\overset{N}{\underset{i=1}{\sum }}v_{i}}Y_{COG}=\frac{\overset{N}{\underset{i=1}{\sum }}\left(y_{i}•v_{i}\right)}{\overset{N}{\underset{i=1}{\sum }}v_{i}}Z_{COG}=\frac{\overset{N}{\underset{i=1}{\sum }}\left(z_{i}•v_{i}\right)}{\overset{N}{\underset{i=1}{\sum }}v_{i}} \end{array}$$

Equation 7: Center of Gravity Formula for three dimensions where x_i_ are the x positions of the dataset, y_i_ are the y positions of the dataset, z_i_ are the z positions of the dataset, v_i_ are the values of the dataset, and N is the total number of points in the dataset.

The position of the center of gravity is given by Equation 7. The COG's position is reported as distance, in units of the scan resolution, from the reference point as mentioned in the preface of this chapter.

#### Peak

The position of the peak, or absolute maximum, value of the fitted data function is also reported. Although not as represented in the neuroscience literature, its use as a measurement may be of interest to some users. The peak position is given as the point in 3D space at which the highest value of the fitted data function occurs. Like the other measurements of position it is reported as distance, in units of the scan resolution, from the reference point.

#### Surface Area

Surface area is computed using a discrete integration method shown in equation 8. The surface area is dependent on a threshold value that is set to 0 at default.

$$\begin{array}{l} SA=\overset{N}{\underset{i=1}{\sum }}||p_{i}\times q_{i}|| \end{array}$$

Equation 8: SA is the surface area value. p_i_ and q_i_ are the vectors pointing in the θ and ϕ directions. N represents the total number of positions whose values are above the threshold.

Consider one position on the motor map whose value is above the threshold. This position is described by two angular coordinates (θ,ϕ). Now, we define three points: (θ,ϕ), (θ+s,ϕ), (θ,ϕ+s) where s is the value of the pixel spacing of the topographic map (in radians). These three points are converted to 3D Cartesian coordinates using the fitted ellipsoid discussed previously. Now, we define a vector *p* between two 3D coordinates corresponding to (θ,ϕ) and (θ+s,ϕ). Then, we define a vector *q* between two 3D coordinates corresponding to (θ,ϕ) and (θ,ϕ+s). The norm of the cross product between these two vectors represents the surface area of the patch defined by that position on the topographic map. This process is then repeated for all positions on the topographic map whose values are above the threshold.

The COG is a weighted average of the motor map's position, and represents the center of the target muscle representation. This measure is biased by design toward the highest recorded MEPs (which also are less variable; Pellegrini et al., 2018), and is a historical and current standard reporting parameter (Wilson et al., 1993; van de Ruit and Grey, 2015). Surface area, however, has no bias and counts all area that is higher than the chosen threshold equally.

#### Volume Integral

Volume integral is computed similarly to surface area as shown in Equation 9.

Volume integral gives the volume under the curve of the motor map function by summing the volumes of patches whose intensity is higher than the threshold. Its units are (map unit)^*^(scan resolution unit)^2^.

$$\begin{array}{l} VI=\overset{N}{\underset{i=1}{\sum }}||p_{i}\times q_{i}||•v_{i} \end{array}$$

Equation 9: VI is the volume integral value. p_i_ and q_i_ are the vectors pointing in the θ and ϕ directions. v_i_ represents the intensity of the motor map. N represents the total number of positions whose values are above the threshold.

This measurement can be described as the sum total of all μV per area within the sampling field and thus is useful for estimating the overall excitability of the cortex. It differs from surface area in that it is weighted toward the higher value regions. Unlike COG, it reports an intensity so its value will differ if the overall excitability of the cortex decreases. For example, if one were to consider two motor maps that were identical except that every likewise point in the first was twice that of the other, the COG of both would be the same, but the VI of one would be double the other.

### Comparing Motor Maps

A key feature of NeuroMeasure is the ability to compare values between datasets. NeuroMeasure was designed to handle both temporal and spatial analysis of maps within the same patient only. There are two ways to compare motor maps within a patient; the first is through direct comparison of numerical values computed from continuous data type motor maps, and the second is through predictive analysis with a motor map that acts as a temporally predictive model of categorical data. The second is much more limited in scope than the first, but both are discussed below.

#### Continuous Data Comparison

**Figure 6:**
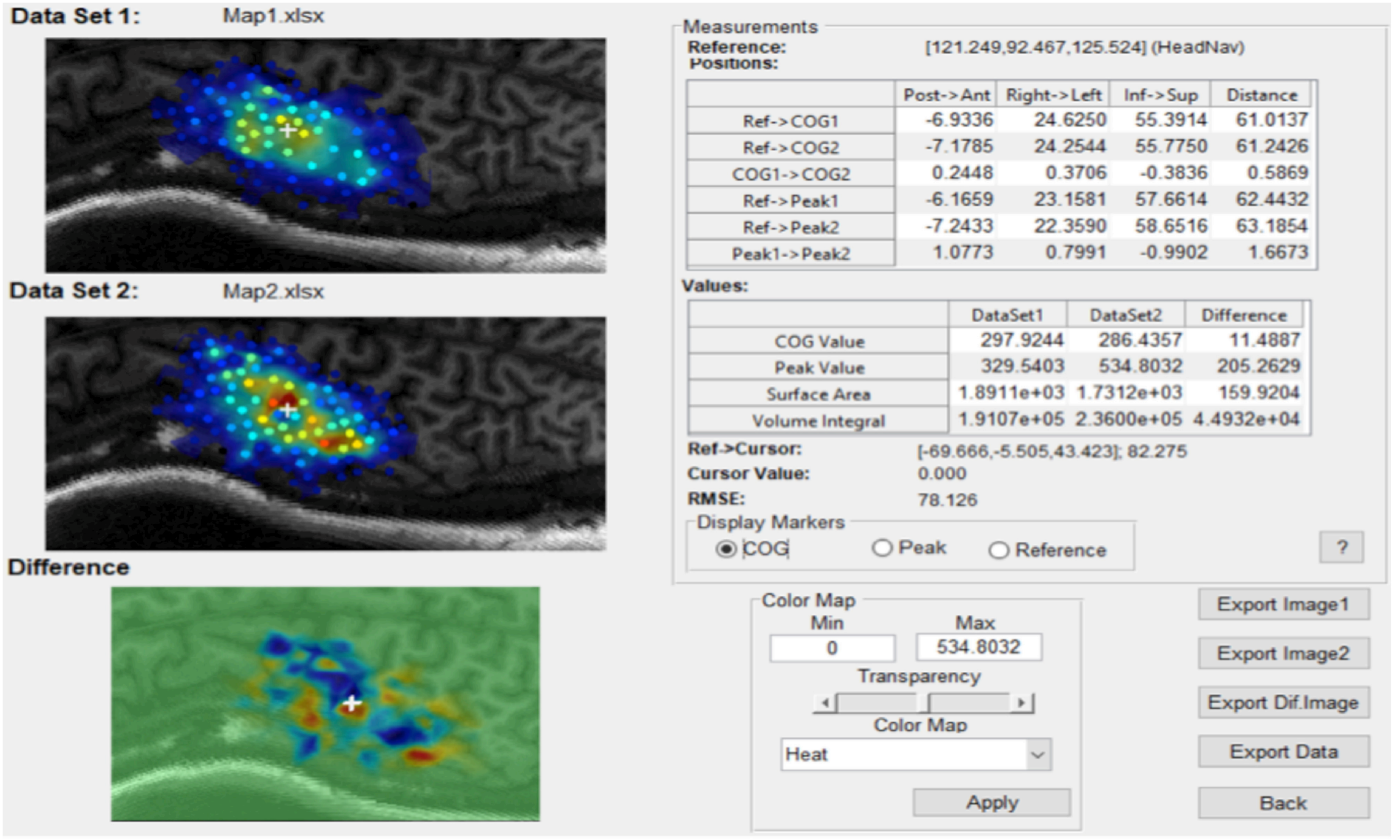
An example of the comparison window launched in continuous comparison mode. The reference point coordinates (in their respective coordinate system) is displayed on top and all position measurements are reported as distance between combinations of two points.

For any continuous dataset NeuroMeasure will fit both data with one selected fitting algorithm and display both maps and their difference, illustrated in Figure 6. The *Measurements* panel features not only single map information present in the main GUI window, but also metrics related to the difference, such as the change in center of gravity location, change in peak MEP location, and surface area/volume integral difference. Continuous motor map comparison is recommended for comparing motor maps taken over time and between the left and right hemisphere.

The root mean square error (RMSE) is also available and displayed at the bottom of the *Measurements* panel in Figure 6. The root mean square error is computed as shown in Equation 10.

RMSE can be interpreted as the average difference between every set of two likewise points on the two motor maps. Note that RMSE is relevant only when the two maps being compared are sampled in the same region of the brain, otherwise there are no likewise points to compare.

$$\begin{array}{l} RMSE=\sqrt{\frac{\overset{N}{\underset{i=1}{\sum }}{\left(P_{i}-O_{i}\right)}^{2}}{N}} \end{array}$$

Equation 10: RMSE is the root mean square error value. n represents the total number of data points. O_i_ represents the ground truth value of one raw data point from data set 1. P_i_ represents the predicted value from data set 2's motor map function at the same location as O_i_.

#### Categorical Data Comparison

The categorical data comparison window is launched when the clustering consolidation method is set to *probability* and has only one specific usage: to quantify the stability of a motor map reading over time. When set to *probability*, the Compare button will compute the receiver operator curve (ROC) in a procedure outlined below.

**Figure 7:**
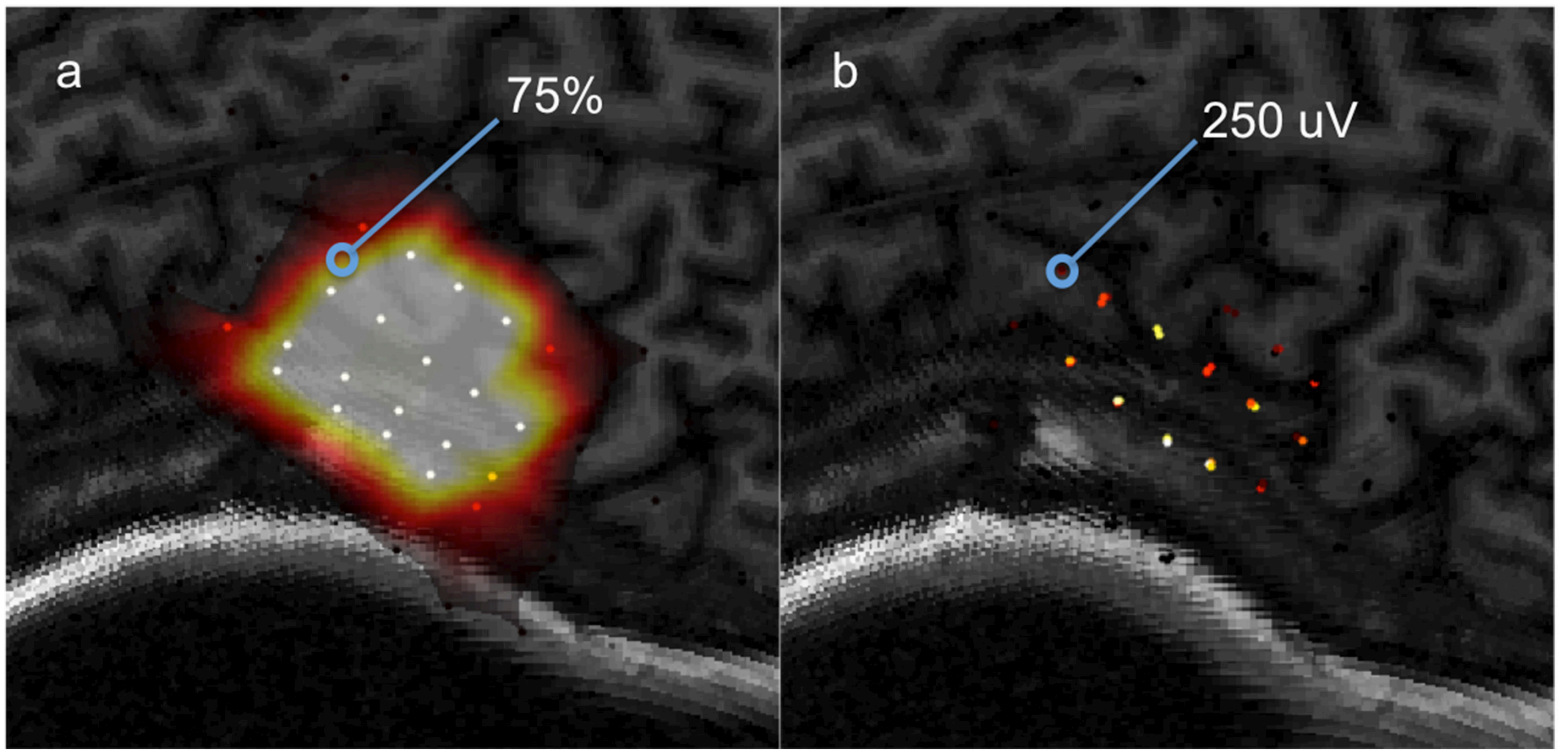
A visual aid for computing ROC between a predictive model and testing data, **(a)** a predictive model computed from a training set, **(b)** the testing set used to evaluate the model's predictions.

A probabilistic model is first generated by fitting data clusters consolidated using the *probability* procedure discussed previously, to a surface function. The resulting model predicts at every location a probability (from 0 to 100%) that a value above the set binarization threshold will be elicited by the TMS pulse. This leads to a motor map shown in in Figure 7. The dataset used to generate this predictive model will be referred to as the “training set.”

**Figure 8:**
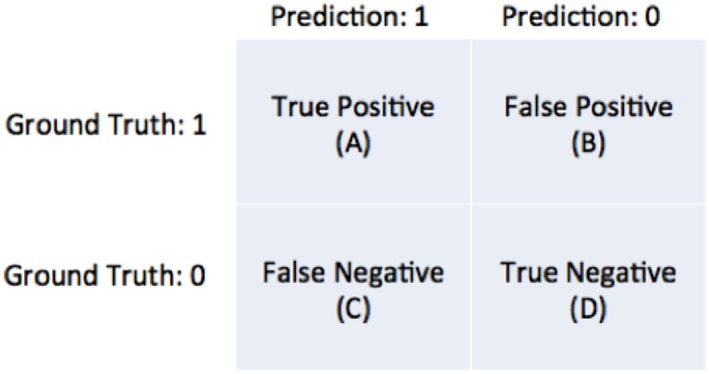
Diagram of the four possible outcomes of a model's prediction tested against a ground truth.

The predictions of the model are tested using a “testing set” of data selected by the user. Consider one data point from the testing set to be 250 μV and the model's prediction for that location is 75%. Now, assume that the binarization threshold used to binarize our training data was 100 μV. Thus, 250 μV is higher than 100 μV so this testing point has a value of 1. Now, we define an arbitrary cutoff threshold in the range of 0–100% that binarizes 75% to either a 0 or 1, and the model's prediction is checked with the testing point. There are four possible outcomes illustrated in Figure 8: the model and testing point are both 1 (true positive), the model predicts 0 but the testing point is 1 (false negative), the model predicts 1 but the testing point is 0 (false positive), and both the model and testing point are 0 (true negative). This is repeated for all points in the testing set. Now, we can compute the true positive rate and the false positive rate using Equation 11a/b.

**Figure 9:**
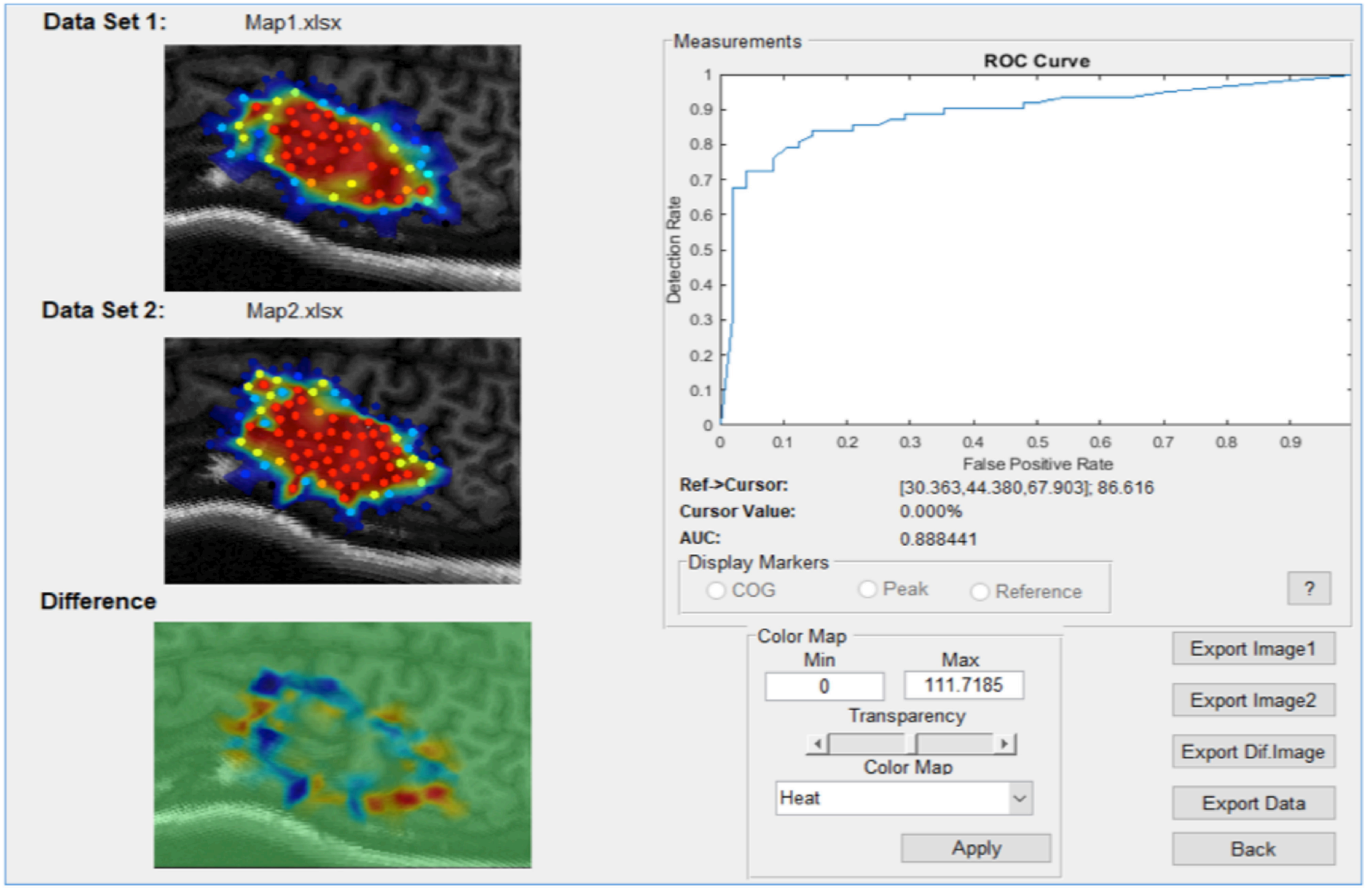
An example of the comparison window launched in categorical comparison mode.

Now, we compute the true positive rate (also known as detection rate) and false positive rate for a sequence of arbitrary cutoff thresholds varying from 0 to 100%. We can then plot the false positive rate against the detection rate for each of the map thresholds in the sequence to generate the curve shown in in Figure 9. This is the ROC and the area underneath the curve (AUC). This is a metric used for quantifying the performance of the predictive model. A model that has an AUC of 1 always predicts correctly with respect to the testing set (this is the best score). A model with an AUC of 0 is one that always predicts the opposite of the testing set (this is still perfect performance, just inverted). A model with an AUC of 0.5 is randomly guessing with respect to the testing set (this is the worst score).

$$\begin{array}{l} TPR=\frac{A}{A+C}\;FPR=1-\frac{D}{D+B} \end{array}$$

Equation 11a/b: Formula for true positive rate (TPR) and false positive rate (FPR). A is the number of true positives, C is the number of false negatives, B is the number of false positives, D is the number of true negatives.

In the case of motor mapping, the AUC is a good way of testing the stability of a motor map over time. By computing the AUC, we test the following hypothesis: How well does a motor map that predicts the likelihood of a TMS response above a specified value threshold perform when evaluated on test data collected at a different time point under different experimental conditions? The more consistent the testing set is with the training set, the closer the AUC will be to 1. Conversely, the more inconsistent the measurements are, the closer the AUC will be to 0.5. Notably, the binarization threshold is an important part of this analysis, and we do not currently have recommendations on its use however this is an area of future work. This functionality of NeuroMeasure at the time of publication is experimental and has no precedence in the literature, unlike the continuous comparison measurements.

### Case Examples

Now, we use a case**-**based approach to highlight the advantages and disadvantages of our software in interpreting the results of some exampled motor mapping experiments. We encourage readers to download the data used in these examples from our Github and follow the workflow, if interested. An operation manual is also available for download for clarification on the settings used in the below examples.

#### First-Sampling vs. Second-Sampling Comparison

In this first case, we use the application to analyze the results of an experiment testing the stability of MEP measurements. The data was collected with the Nexstim neuronavigation platform on a healthy subject with a 0.5 × 0.5 cm grid using an established protocol (Kohl et al., 2006). Immediately afterwards, the same region was sampled a second time with a new 0.5 × 0.5 cm grid offset from the first by 0.25 cm so that each sample point was roughly between the sampling of the first grid. Three samples per location were collected. The highest recorded single MEP was 671 μV peak-to-peak and the highest average of three was 549 μV. The datasets used for this example are shown in Figure 1.

After clustering the repeated measures with a QTC threshold of 0.02 (found by trial and error), the clustered subsets are averaged to single representative values and fitted to a surface function using the Piecewise Cubic algorithm. A comparison is than made between the first dataset and the second shown in Supplemental Figure 1A. The results show that the COG has shifted 2.5 mm to the left posterior and the peak average of three measurements has shifted 13.9 mm to the left posterior. Being a weighted average, COG tends to fluctuate less than the peak as is demonstrated here. The difference in the surface area (computed with a threshold of 0 μV) is 39.98 mm^∧2^ and the difference in volume integral is 3.349e+04 mm^∧2^^*^μV. The RMSE, or average error between any two given points in the field, is 75.395 μV.

After applying the probability consolidation method with a binarization threshold of 100 μV and relaunching the comparison, we arrive at the window shown in Supplemental Figure 1B. The system reports the receiver operator curve generated from our model fit to dataset 1 attempting to predict the values in dataset 2. Since the two maps under comparison are expected to be similar, we expect the AUC to indicate good performance (close to 1). At 0.93, that is the case. The ROC will be close to a square curve because there are one or more thresholds that create optimal performance. Of course, the AUC is highly contingent on the binarization threshold used to compute the cluster probabilities, which is a limitation of this analysis and a subject of future work.

#### Pre-fatigue vs. Post-fatigue Comparison

This example is very similar to the previous example (First Sampling vs. Second Sampling Comparison), with the exception that we use data from a different experiment. In this experiment, the motor cortex was sampled in a 1 × 1 cm grid using the BrainSight system, prior to a hand fatigue exercise (pre), then sampled again immediately afterwards (post), and then sampled again 60 min after that (post+60). Four samples were collected per stimulation site. The data is displayed in Supplemental Figure 2. Fatigue motor exercise is well documented in the literature to change the excitability of the motor cortex, and we expect that the pre vs. post-motor maps will be less similar than the pre vs. post+60 motor maps (Chye et al., 2010; Sidhu et al., 2018).

After uploading all three datasets to NeuroMeasure and clustering the repeated measures with a QTC threshold of 0.02, the data is consolidated using the averaging method and fitted to a surface with the Piecewise Cubic algorithm. First, we launch a comparison between the pre-fatigue and post-fatigue data shown in Supplemental Figure 3A. We can see that the COG has shifted 3.9 mm to the right anterior, the peak has shifted 9.9 mm to the right posterior, the motor map's surface area above 0 μV has increased by 932 mm^∧2^ and the volume integral representing overall cortex excitability has risen by 2.032e+05 mm^∧2^^*^μV. Now we launch our comparison between pre and post+60 datasets shown in Supplemental Figure 3B. The COG has shifted 3.3 mm to the right anterior, peak has shifted 14.5 mm to the right anterior, the surface area above 0 μV has decreased by 132.77 mm^∧2^ and the volume integral has decreased by 6.193e+04 mm^∧2^^*^μV. These measurements tells us that that the surface area of motor cortex involvement rose post-fatigue by almost an order of magnitude and then fell after 1 h back to baseline. The same can be said of overall cortical excitability as evident by the VI. The RMSE of pre vs. post, 1406.12 μV is more than double that of pre vs. post+60 at 602.55 μV.

If we relaunch the comparison of pre vs. post in categorical mode using the probability consolidation method, the window appears as in Supplemental Figure 3C. As we can see by the AUC of 0.8, the model fit onto the pre dataset is a poor predictor of the post-dataset. Now looking at the pre vs. post+60 comparison shown in Supplemental Figure 3D, the AUC is now 0.99 meaning the model fitted to the pre dataset is very good at predicting the post+60 measurements. As mentioned previously, the binarization threshold used to calculate the probabilities will vastly change the analysis, and the one used for this example was arbitrarily chosen to be 3,000 μV. At a binarization threshold of 1,000 μV, the AUC's for pre vs. post and post+60 are both 0.9 further demonstrating that standardizing the binarization threshold will be an important part of using this analysis.

#### Left vs. Right Hemisphere Comparison

In this example we demonstrate NeuroMeasure's capability of comparing motor maps collected on the right and left hemisphere of the same subject and comparing the map features between them. Notably the dataset presented in this example is fabricated for the purpose of demonstration. Real measurements could not be acquired so simulated data was used instead.

The resulting comparison window is shown in Supplemental Figure 4. The right hemisphere COG is 5 mm more right from the midline than the left hemisphere COG is to the left. The right hemisphere COG is also 2.65 mm more posterior than the left. The SA of the right hemisphere is 344 mm^∧2^ less than the SA on the left when 0 μV is used as the inclusion threshold. The VI is 1.589e+05 mm^∧2^^*^μV greater on the left than on the right indicating that the right hemisphere is more excitable. Notably, the RMSE is meaningless in this analysis since the two maps are not spatially overlapping and therefore have no measurements in common. The same can be said of ROC analysis.

#### Cerebral Palsy Peak Discretization

Sometimes, motor mapping data from subjects with neurologic conditions can yield uncharacteristic data like that shown in Supplemental Figure 5A. This dataset is recorded from a subject with cerebral palsy. Note that this dataset contains many individual “hotspots,” or relative peaks surrounded by lower MEP values. In some experimental protocols or data analysis regimes, it may be useful to separate, or discretize the motor map into individual peaks and calculate measurements for those portions alone. With this in mind, NeuroMeasure comes with an automated peak discretization feature capable of doing so.

Supplemental Figure 5B shows the discretization feature enabled. A hotspot has been singled out from the total motor mapping field. Its COG, peak, SA, and VI are calculated with only the data points within the marked white boundary. The user has some control over the inclusion boundary so it can be customized to include certain regions of the mapping field but not others.

## Discussion

The advent of neuronavigation has ushered a new world of possibilities in studying the neurophysiology of the brain and its ever shifting architecture. Use of this technology has already generated many findings (Lefaucheur and Picht, 2016; Lüdemann-Podubecká and Nowak, 2016) and many more are sure to come. Here, we present a software tool in an effort to make neuronavigation-based neuroscience research more accessible by providing a set of graphical and statistical tools with which to visualize and analyze data. The choice of tools and visuals was informed by some notable challenges in the neuroscience field concerning the non-stationary nature of MEP data, the sampling limitations of TMS and the lack of standardization in data collection. With this tool we seek to make motor mapping data easier to characterize and compare longitudinally. Through the use of model fitting, we hope to overcome TMS sampling limitations and the lack of data standardization by using interpolated values to be able to make pairwise coordinate comparisons between motor maps when the sampling pattern does not align. Furthermore, access to measurements like surface area and volume integral, and comparative metrics like RMSE and AUC, could have advantages over COG in understanding the phenomenon of neuroplasticity. Finally, the software seeks to build an intuitive framework for tracking changes in these metrics within patients over time by reporting measurements of position with respect to a standardized reference point selected by the user, and being flexible to a range of experimental protocols, including multiple stimulation vs. single stimulation sampling arrangements by supplying tools for repeated measures data clustering.

NeuroMeasure is not a comprehensive tool and it has its limitations. The core of our approach in fitting TMS data to surface functions that are then sampled to make likewise positional comparisons in other data sets essentially lacks validation. It is not known how changes in the TMS sampling spacing change the integrity of interpolated values nor if a sampling limit exists beyond which this approach is no longer valid. Furthermore, while we provide a range of surface fitting algorithms to choose from, which algorithm is best suited for a particular purpose remains unexplored. The de-dimensionalization procedure currently imposes some distortion to the original positions of data points, as previously mentioned, so a better procedure is a subject of future work. Finally, while the structure of the workflow is suited for making within-patient comparisons, it is not currently possible to make comparisons between patients. Such a procedure would likely require a form of image registration between head MRI's in order to establish a shared coordinate space in which distances can be reported normalized to inter-patient anatomy.

## Author Contributions

MG, AM, SS, AC, and UA: software development, methods development, and manuscript preparation; GT and AK: methods development, and manuscript preparation; JS and KT: data and manuscript preparation; KF, TS, and DE: data preparation, methods development, and manuscript preparation.

### Conflict of Interest Statement

The authors declare that the research was conducted in the absence of any commercial or financial relationships that could be construed as a potential conflict of interest.
