# Supplementary material for: NeuroMeasure: A Software Package for Quantification of Cortical Motor Maps Using Frameless Stereotaxic Transcranial Magnetic Stimulation
Source: Front Neuroinform. 2019 Apr 16;13:23. doi: 10.3389/fninf.2019.00023 (PMC6499165; doi:10.3389/fninf.2019.00023)
Supplement: Supplementary file 1 [file Data_Sheet_1.PDF]

NeuroMeasure: a software package for quantification of cortical motor maps using frameless stereotaxic transcranial magnetic stimulation

Supplementary Material

Supplementary Material 1:

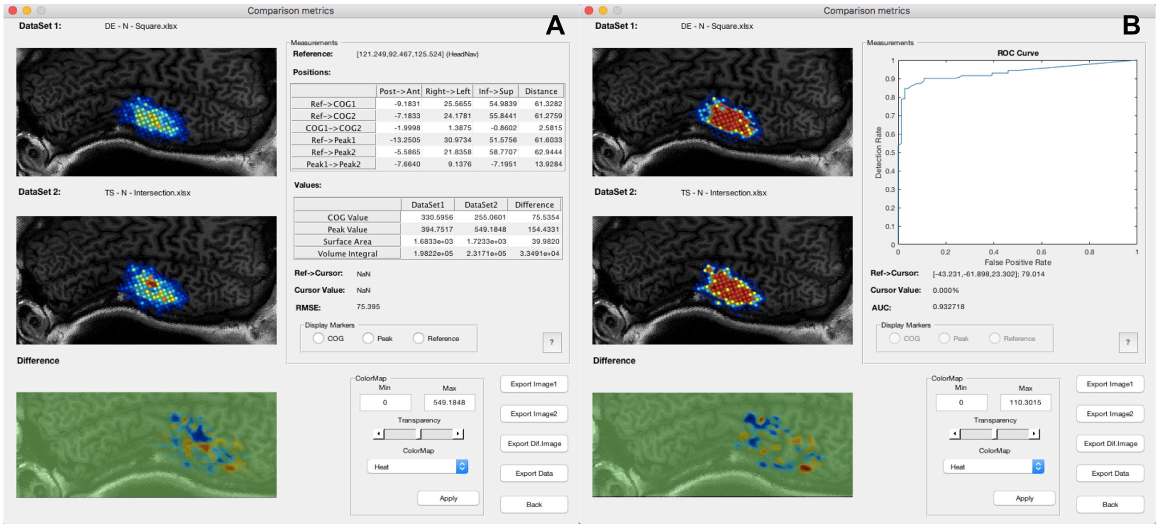

Supplemental Figure 1: NeuroMeasure's comparison window launched in continuous mode (A) and in categorical mode (B) for the analysis of datasets shown in Figure 1.

Supplementary Material 2:

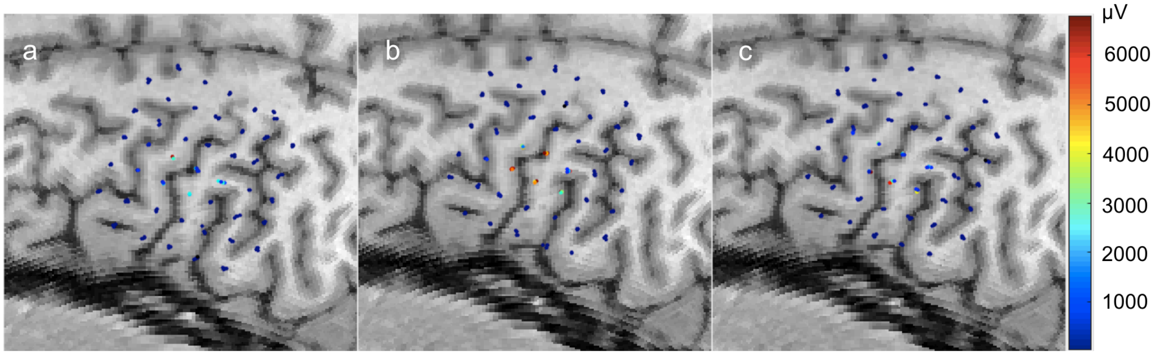

Supplemental Figure 2: Individual motor evoked potential amplitudes sampled four times per location in a 1x1 cm gri and were color categorized. Recorded from the FDI muscle with TMS stimuli delivered over the dominant (left) hemisphere and registered to MRI using BrainSight neuronavigation system. a) Sampling prior to hand fatigue exercise, v) sampling immediately after hand fatigue exercise, c) sampling 60 minutes after hand fatigue exercise.

Supplementary Material 3:

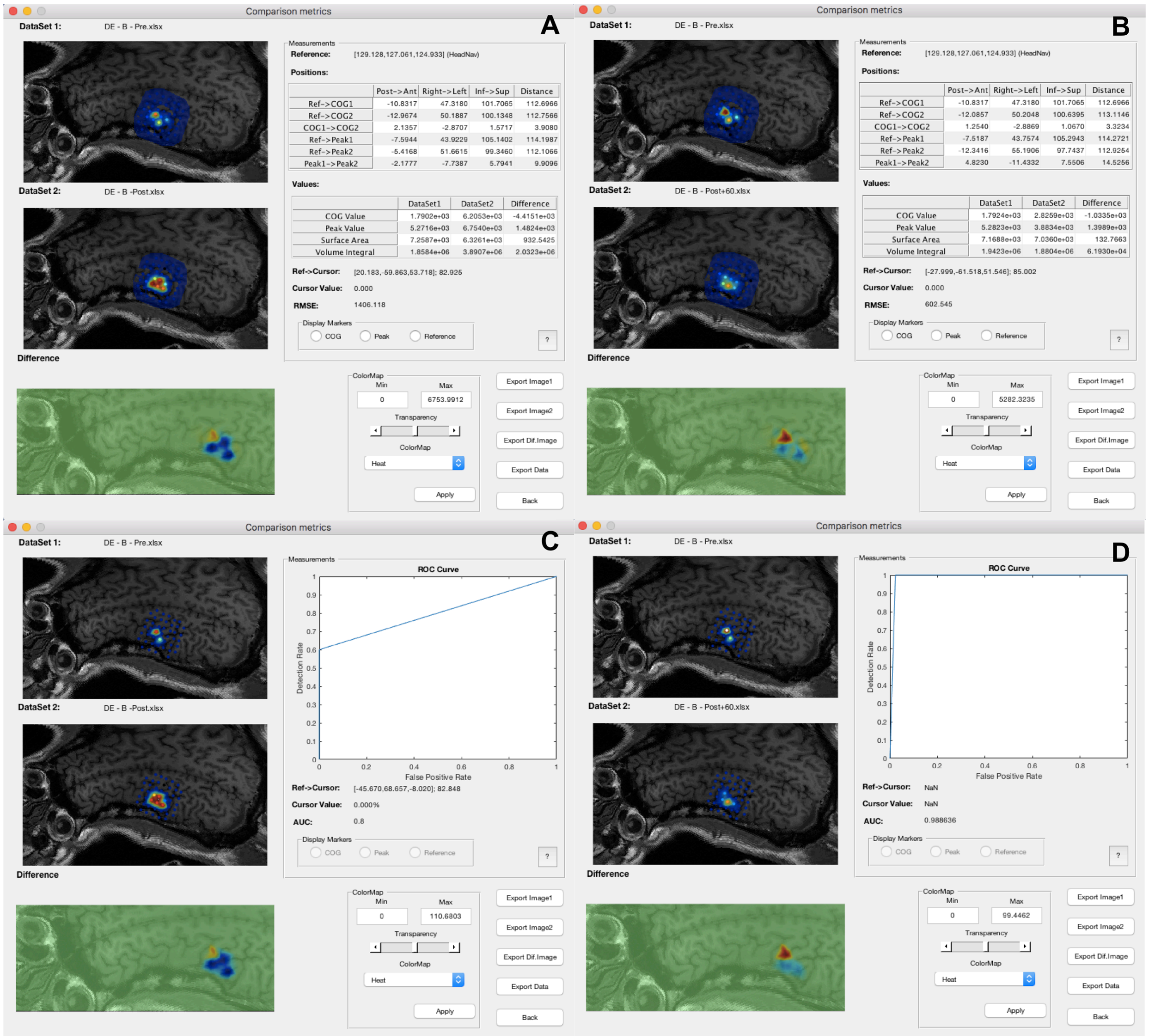

Supplemental Figure 3: The analysis of the pre-fatigue/post-fatigue data shown in Supplemental Figure 2. A) Continuous mode comparison of pre vs post, B) Continuous mode comparison of pre vs post+60, C) Categorical mode comparison of pre vs post, D) Categorical mode comparison of pre vs post+60.

Supplementary Material 4:

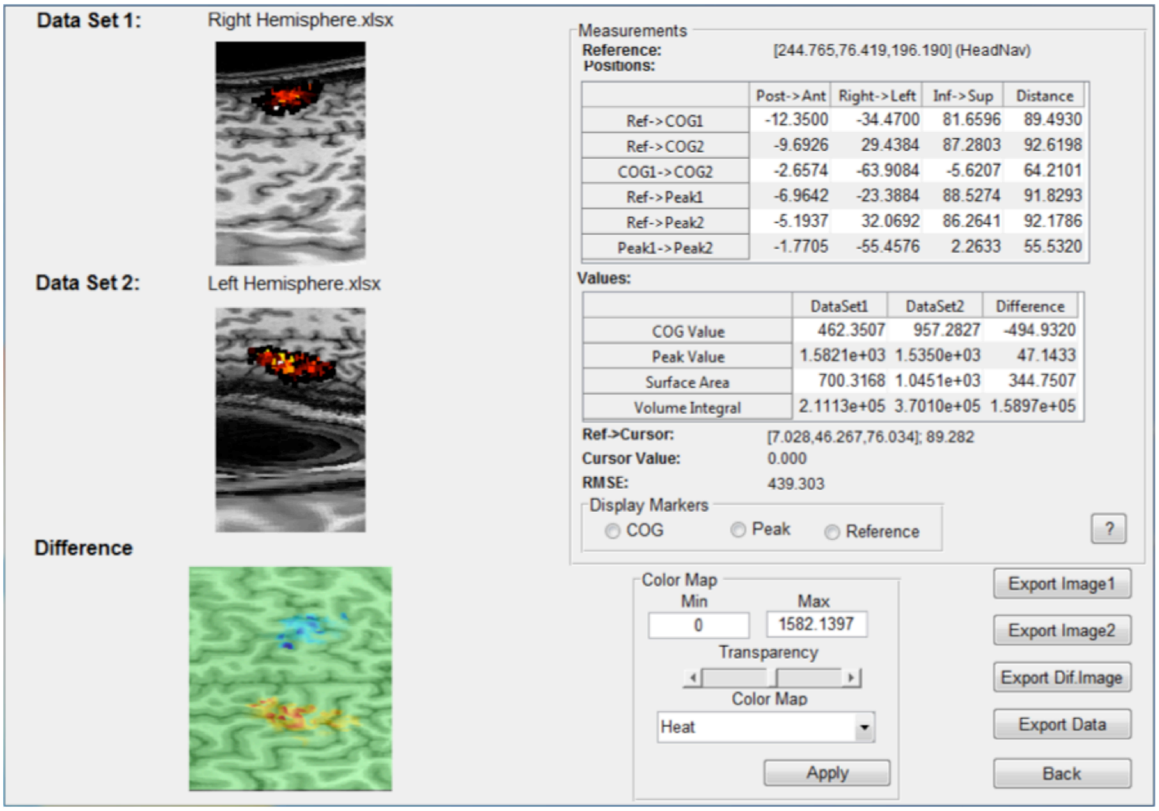

Supplemental Figure 4: An example of the comparison window when launched to compare data sets on the left and right hemisphere. When the motor maps are not overlapping the difference map subtracts non-zero values from a field of zeros to produce the exampled display. RMSE, in the absence of likewise values, simply represents the average value of data set 1.

Supplementary Material 5:

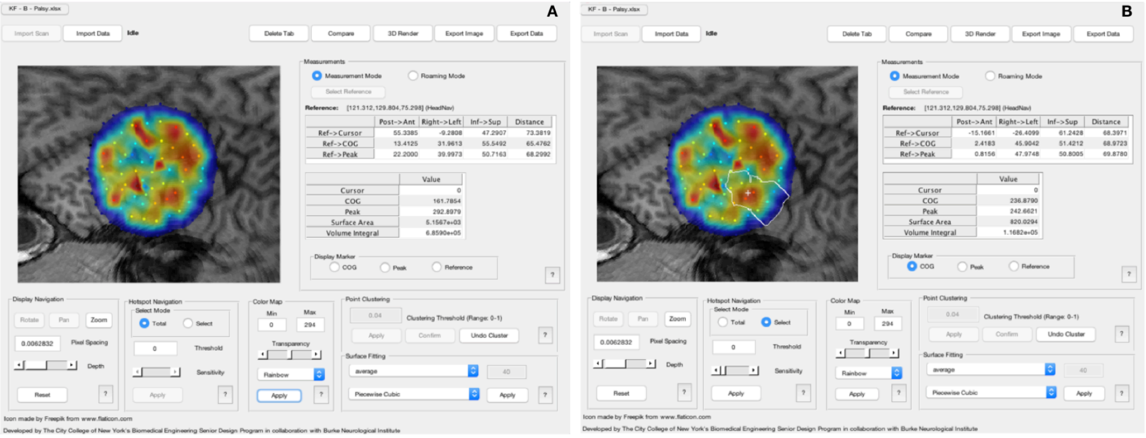

Supplemental Figure 5: A) The NeuroMeasure main window showing an example of a cerebral palsy motor map that has been clustered and surface fitted. B) The same motor map displaying NeuroMeasure's peak discretization feature.
